# Lassa Fever, Nigeria, 2003 and 2004

**DOI:** 10.3201/eid1110.041343

**Published:** 2005-10

**Authors:** Sunday Aremu Omilabu, Sikiru Olanrewaju Badaru, Peter Okokhere, Danny Asogun, Christian Drosten, Petra Emmerich, Beate Becker-Ziaja, Herbert Schmitz, Stephan Günther

**Affiliations:** * College of Medicine of the University of Lagos, Idi-Araba, Lagos, Nigeria; † Irrua Specialist Teaching Hospital, Irrua, Edo, Nigeria; ‡ Bernhard-Nocht Institute for Tropical Medicine, Hamburg, Germany

**Keywords:** Lassa fever, Nigeria, arenavirus, letter

**To the Editor:** Suspected outbreaks of Lassa fever have been reported in the northern part of Edo, Nigeria, including Ekpoma, Igarra, and Ibilo, in 2001 and between November 2003 and March 2004 (*1**,**2*). To confirm Lassa fever activity in this area, serum samples were collected at the Specialist Teaching Hospital in Irrua (ISTH) from September 2003 to January 2004. Approximately 16,000 patients are seen each year at ISTH, and ≈80% of them have febrile illness. Serum specimens were taken from patients with febrile illness (n = 31), healthy contact persons (n = 17), and healthy hospital staff (n = 12). The samples were analyzed by Lassa virus–specific reverse-transcriptase polymerase chain reaction (RT-PCR) at the University of Lagos. Aliquots of specimens were sent to the Bernhard-Nocht Institute (BNI in Hamburg, Germany) for confirmatory PCR analysis, serologic testing, and virus isolation. The PCR used at both facilities was based on primers 80F2 and 36E2 that targeted the glycoprotein precursor (GPC) gene (*3*), although the protocols were slightly different. At BNI, virus RNA was purified by QIAamp viral RNA kit (Qiagen, Hilden, Germany), and RT-PCR was performed with Superscript II RT/Platinum Taq polymerase 1-step reagents (Invitrogen, Karlsruhe, Germany). This PCR assay has a 95% detection limit of 2,500 copies/mL (*4*). At the University of Lagos, virus RNA purification and RT-PCR were performed with diatomaceous silica and Brilliant single-step RT-PCR kit (Stratagene, Heidelberg, Germany), respectively. Serologic testing for Lassa virus–specific immunoglobulin G (IgG) and IgM was performed by indirect immunofluorescence assay (IFA) by using Vero cells infected with Lassa virus strain Josiah. Virus was isolated in the biosafety level 4 laboratory at BNI with Vero cells. Results of the tests are summarized in the Table.

**Table:** Lassa virus–specific findings in 60 serum samples from Irrua Specialist Teaching Hospital, Edo, Nigeria*

Patient	RT-PCR†	IgM titer‡	IgG titer‡
Patients with fever (n = 31)			
04-10	Positive§	–	–
04-02	Positive	1:40	–
04-51	–	1:160	–
04-34	–	1:40	–
04-03	–	1:>20,480	1:20,480
03-05	–	1:320	1:20,480
03-01	–	1:160	1:10,240
04-08	–	1:80	1:20,480
04-33	–	1:20	1:640
04-52	–	1:160	1:40
04-53	–	1:40	1:40
Contact persons (n = 17)			
04-04	Positive	1:20	–
03-04	–	1:160	1:>20,480
04-11	–	–	1:80
Hospital staff (n = 12)			
04-31	–	–	1:80
04-32	–	–	1:80
04-17	–	–	1:80
04-20	–	–	1:20

*Data not shown for patients whose samples were negative in all tests. RT-PCR, reverse-transcriptase polymerase chain reaction; Ig, immunoglobulin; –, negative result. †RT-PCR targeting the Lassa virus glycoprotein gene (*4*). PCR products were detected in ethidium bromide–stained gel and sequenced (GenBank accession nos. DQ010030 and DQ010031 for 04-02 and 04-10, respectively). ‡Immunofluorescence assay used cells infected with Lassa virus strain Josiah. Findings were confirmed with μ-capture and IgG enzyme-linked immunosorbent assays (data not shown). §Lassa virus was isolated in cell culture (strain Nig04-010), and part of the L gene was sequenced (GenBank accession no. AY693637).

Acute Lassa virus infection, as shown by a positive PCR result, was diagnosed at the University of Lagos in 1 patient. This result was independently confirmed at BNI, and 2 additional samples tested positive by PCR. The PCR signals were weak, which suggests that discrepancies between laboratories stem from higher sensitivity of the assay used at BNI. Presence of a low IgM titer in the absence of IgG in 2 of the PCR-positive samples is also consistent with an acute infection. Two of the Lassa virus–positive persons (04-02 and 04-10) had febrile illness that indicated symptomatic Lassa fever, while 1 (04-04) had been classified as an asymptomatic contact at the time of sampling. Retrospective investigation showed no evidence of illness in this person before or after sampling. Sequencing the diagnostic PCR fragments (300 nucleotides [nt] of GPC gene) from the 3 patients indicated infections by closely related strains. The sequence of patient 04-10 (GenBank accession no. DQ010031) differed by 4% from those of patients 04-02 and 04-04, while the latter sequences were identical (GenBank accession no. DQ010030). The facts that patients 04-02 and 04-04 were sisters who lived in the same house, that their samples were taken on the same day (January 28, 2004), and that the sequences were identical suggest a common source of infection or an infection chain. The detection of an asymptomatic or mild Lassa virus infection in the contact person agrees with population-based studies in Sierra Leone that show only 9%–26% of all Lassa virus infections are associated with fever (*5*).

In an additional 10 samples, IgM with or without IgG was detected, primarily in patients with febrile illness. IgG in the absence of IgM was detected in 1 contact and 4 healthcare workers. All serologic IFA findings were confirmed with μ-capture and IgG enzyme-linked immunosorbent assays developed at BNI. Virus isolation was attempted with all samples that tested positive by PCR or IgM IFA. Lassa virus was isolated from 1 PCR-positive serum (04-10). The strain was designated Nig04-010. To characterize Lassa virus circulating in north Edo, phylogenetic analysis was performed. In addition to the GPC sequences of the diagnostic PCR fragments, part of the L gene of Nig04-010 was amplified and sequenced (780 nt, GenBank accession no. AY693637). Phylogenetic analysis of these sequences showed that the virus circulating around Irrua belongs to phylogenetic lineage II, which comprises Lassa virus strains from the southeastern part of Nigeria (*6*). Thus, genotype and geographic origin of the viruses characterized here correspond.

These data provide evidence for Lassa fever activity in north Edo. Approximately 6% of febrile patients tested had PCR-confirmed Lassa fever, which extrapolates to hundreds of patients with Lassa fever per year, when one considers the number of patients with febrile illness seen at ISTH. As shown here and elsewhere, PCR is a useful tool to diagnose Lassa virus infection (*3**,**7*), a prerequisite for effective ribavirin treatment (*8*). First steps have been made to establish molecular diagnostics for Lassa virus at the University of Lagos. Further efforts are necessary to improve the laboratory infrastructure in the country.
